# PalmNeXt: a ConvNeXt-based deep learning model for pest detection in date palm leaves

**DOI:** 10.3389/fpls.2025.1738129

**Published:** 2026-01-22

**Authors:** Mahmood Ashraf, Muhammad Zeeshan Aslam, Natasha Saeed, Syed Jawad Hussain

**Affiliations:** 1 Department of Computer Science and Artificial Intelligence, College of Computer Science and Engineering, University of Jeddah, Jeddah, Saudi Arabia; 2 Department of Computer Science, Sir Syed CASE Institute of Technology, Islamabad, Pakistan

**Keywords:** automated pest detection, ConvNeXt-Tiny, data preprocessing, date palm leaves, transfer learning

## Abstract

Automated pest detection is essential for timely and accurate crop monitoring, yet many existing approaches rely on manual inspection or computationally heavy models that struggle with small and variable datasets. To address these challenges, we introduce an enhanced ConvNeXt-Tiny–based framework that incorporates a tailored preprocessing pipeline to improve feature quality and overall performance. The model is evaluated on an RGB image dataset of 3,000 date palm leaf samples across four classes (Bug, Dubas, Healthy, Honey). Its performance is compared against two custom baselines, CNN-Attention and ResNet13-Attention, as well as state-of-the-art models including ViT, ECA-Net, and the standard ConvNeXt-Tiny. Experimental results show that our preprocessing-augmented ConvNeXt-Tiny achieves the highest accuracy, precision, recall, and F1-score, outperforming both custom and state-of-the-art baselines. These findings demonstrate the effectiveness of the proposed lightweight solution for scalable and high-accuracy pest detection in precision agriculture.

## 1 Introduction

Date palms (Phoenix dactylifera) are incredibly important to both the economy and the environment in arid and semi-arid regions, especially in areas like the Middle East and North Africa. These trees don’t just provide food; they help fight desertification, support ecosystems, and offer economic stability to many communities. As a staple crop, date palms are deeply woven into the culture, livelihoods, and diets of millions of people.

However, like many agricultural crops, date palms face constant threats from a variety of pests. Insects such as the Dubas bug (Ommatissus lybicus), honeydew-producing insects like mealybugs and aphids, and leaf-chewing pests including various types of caterpillars can pose significant risks. These feed on the sap, leaves, or fruit, weakening the trees and making them more susceptible to disease. The sticky honeydew they produce can also encourage the growth of sooty mold, further inhibiting photosynthesis and overall plant health.

If these infestations are not identified and managed promptly, the resulting damage can severely reduce fruit yield and quality, impact tree longevity, and cause major economic losses. Traditional methods often rely on manual inspections, which are time-consuming, labor-intensive, and sometimes ineffective in covering large orchard areas. That’s why spotting these infestations as early and as accurately as possible is essential for sustainable farming. Timely detection enables farmers to take targeted action, whether through biological control methods, environmentally safe pesticides, or improved agricultural practices, thereby reducing the need for widespread chemical use and preserving the health of both the trees and surrounding ecosystems. Different detection methods help minimize the occurrence and severity of outbreaks (Gao et al., 2025).

In recent years, deep learning and image-based technologies have opened new doors for automating this work (Liu et al., 2025). Many previous studies in agricultural pest detection rely on complex model combinations, advanced data augmentation techniques, and extensive preprocessing to achieve high accuracy. These approaches often involve multi-stage pipelines, custom modules, and computationally intensive tuning processes. In this study, we show that simple, lightweight transfer learning models, without any data augmentation can still achieve strong performance, offering a more straightforward and efficient alternative.

Existing deep learning approaches for pest detection, particularly lightweight networks and ConvNeXt-based models still face notable limitations. Many struggle to generalize well when trained on small or imbalanced agricultural datasets, while others require complex feature engineering or large computational resources that hinder real-time deployment in field conditions. These challenges highlight the need for a more efficient yet robust model tailored for practical agricultural environments.

To address this gap, we use an RGB image dataset of 3,000 date palm leaf samples categorized into four classes: Bug, Dubas, Healthy, and Honey. The dataset includes considerable variations in lighting, leaf orientation, texture, and background complexity, making it representative of real-world field conditions and suitable for evaluating model robustness.

Our study introduces PalmNeXt a lightweight, efficient deep learning model built on the ConvNeXt-Tiny architecture. By using transfer learning, we fine-tuned the model on a set of labeled RGB images of palm leaves, grouped into four categories: Bug, Dubas, Healthy, and Honey. PalmNeXt is designed to understand both small details and larger patterns in the images, which helps it make accurate predictions even when the leaves are photographed under different conditions.

The results are promising. PalmNeXt not only outperforms existing models in terms of accuracy, but it also runs efficiently, making it a strong candidate for real-time use in smart farming systems. With its balance of performance and speed, PalmNeXt brings us one step closer to scalable, automated pest detection that can help protect crops and support more sustainable agriculture. The key contributions of our research are given below:

- We develop PalmNeXt, a lightweight ConvNeXt-Tiny-based deep learning model specifically optimized for pest detection in date palm leaves, eliminating the need for handcrafted features or hybrid complexity.
- The model is trained on a publicly available RGB image dataset consisting of four pest-related and healthy categories under real-world field conditions.
- We conduct a comprehensive evaluation using class-wise metrics and confusion matrix analysis, demonstrating consistent superiority of our model across all pest categories.

The structure of the paper is organized as follows: Section 2 reviews related work on pest detection. Section 3 discusses the complete methodology of this study. Section 4 outlines the experimental results. Section 5 concludes the study and highlights directions for future research.

## 2 Literature review

The Red Palm Weevil (RPW), Rhynchophorus ferrugineus, is recognized as one of the most destructive pests affecting palm trees globally, posing a serious threat to agricultural sustainability and economic productivity. Early detection is critical to prevent irreversible damage; however, conventional visual and auditory inspection methods are often inadequate during the initial stages of infestation (Arasi et al., 2024). To overcome the limitations of traditional RPW detection methods, recent research has focused on automated, intelligent systems. The IRPWD-BSADL framework combines bilateral filtering, ShuffleNet, BSA, and XGBoost, achieving 99.43% accuracy and outperforming Faster CNN (99.03%) and RPWE-GTODL (99.27%). Another effective model, RPWD-GTODL (Albraikan et al., 2023), employs Gabor filtering, Mask R-CNN with MobileNetV2, and the Gorilla Troops Optimizer (GTO) for hyperparameter tuning, reaching 99.27% accuracy. These results highlight the promise of deep learning integrated with metaheuristic optimization for early RPW detection. Beyond pest detection, the health and productivity of palm trees are also compromised by various physiological and fungal disorders. However, progress in automated disease classification has been hindered by the lack of diverse, high-quality datasets.

To bridge this gap, the study in (Namoun et al., 2024) introduced a comprehensive image dataset featuring eight distinct types of date palm leaf disorders. The dataset, collected from 10 farms in Madinah, Saudi Arabia, using both smartphones and DSLR cameras under diverse lighting conditions, consists of 3697 augmented images. It encompasses physiological deficiencies (e.g., potassium, manganese, and magnesium), fungal infections (e.g., black scorch, leaf spot, fusarium wilt, rachis blight), pest-related disorders (e.g., Parlatoria blanchardi), and healthy samples. This dataset represents a critical resource for training robust, deep learning-based models capable of distinguishing between various stress factors in palm agriculture. Complementing ground-based image analysis, the integration of Unmanned Aerial Vehicles (UAVs) with remote sensing and object detection models has enabled large-scale palm tree monitoring.

The study published in Neural Networks (Jin et al., 2026) addresses key challenges arising from limited labeled 3D medical images, including weak feature discrimination, overlapping organ boundaries, and poor generalization across varying anatomical structures. A combined multi-modal CT/MRI dataset is used to evaluate the proposed Pseudo-label Enriched Segmentation Framework (PESF). The framework leverages pseudo-label generation, confidence-based filtering, and multi-step refinement to enhance feature separability without requiring additional manual annotation. Overall, PESF strengthens representation learning and improves segmentation robustness under constrained labeling conditions.

A recent study (Hajjaji et al., 2025) used YOLOv8 and YOLOv5 to detect palm trees from UAV imagery, with YOLOv8-HighAug achieving the best performance (AP: 0.88, precision: 0.87, recall: 0.86). This highlights the potential of UAV-based deep learning for real-time plantation monitoring. Additionally, with limited success from chemical methods, mathematical modeling is gaining importance for evaluating integrated RPW control strategies.

In (Alnafisah and El-Shahed, 2024), a dynamic model incorporating mechanical injection, pheromone traps, and the Sterile Insect Technique (SIT) was developed to simulate RPW population dynamics. Using the Forward-Backward Sweep method, the study assessed local stability and bifurcation behavior under varying intervention intensities. Results indicated that mechanical injection, when applied above a critical threshold, significantly reduces larvae and pupae populations, emphasizing the potential of integrated, model-driven control strategies in pest eradication.

Further advancing automated disease detection, a two-stage optimization methodology was introduced in (Savaş, 2024), combining transfer learning with deep ensemble learning. Pre-trained deep neural networks were fine-tuned on palm-specific datasets, and ensemble strategies, particularly the Dirichlet Ensemble Learning Method (DELM1), were employed to boost predictive accuracy. The proposed ensemble model achieved an ROC-AUC score of 99%, outperforming individual base learners and highlighting the value of ensemble-based transfer learning for palm disease classification tasks. Lastly, the need for stage-wise classification of specific disorders such as White Scale Disease (WSD) has driven the adoption of classical machine learning approaches.

In (Hessane et al., 2023), a framework was developed using image features extracted via Gray Level Co-occurrence Matrix (GLCM) and HSV color space. Classifiers such as SVM, KNN, Random Forest (RF), and LightGBM were trained on over 2000 labeled images, including healthy samples and those affected by WSD at different stages. With data augmentation addressing class imbalance, the SVM model using combined GLCM and HSV features yielded the highest accuracy of 98.3%. This emphasizes the potential of hybrid feature-based approaches in precise stage-wise disease classification, facilitating timely and targeted intervention.

To improve small pest detection in high-resolution images, the study in (Chen et al., 2025) proposed the DAMI-YOLOv8l framework. It integrates a Depth-wise Multi-scale Convolution module, ASF-P2 for small-object fusion, and the MPDinner-IoU loss for better localization. Trained on the LP24 dataset, it achieved a mAP@50 of 78.2% and mAP@50:95 of 57.3%, with 121.12 FPS inference speed. Its robustness was also confirmed on Pest24 and VisDrone2019 datasets.

Expanding UAV-based pest detection (Sun et al., 2025), introduced YOLO-UP, an enhanced YOLOv8n model tailored for dense cotton fields. It incorporates SC3, AFPN, LSKA, and GeLU to improve feature extraction and detection under occlusion and clutter. Trained on 2,090 UAV images, YOLO-UP outperformed YOLOv8n and others with a 3.46% mAP@50 increase, and notable gains in precision (5.16%) and recall (7.81%), while remaining lightweight for mobile use. Complementary research has also targeted specific crop types.

In (Zhang et al., 2024), the JutePest-YOLO model is introduced for detecting multiple small pest species within jute cultivation environments. Based on YOLOv7, the model incorporates an enhanced ELAN-P backbone, a P6 detection layer to improve sensitivity to small targets, and the WIoU v3 loss function for more precise bounding box regression. A large-scale jute pest dataset was curated with nine pest classes, supplemented by data augmentation techniques to ensure model robustness. Experimental evaluation yielded a mAP@0.5 of 95.68% and a 16.05% reduction in GFLOPs compared to YOLOv8s, demonstrating superior performance in both accuracy and computational efficiency. Ablation studies further confirmed the significance of each architectural component in improving small-object pest detection.

To support resource-constrained environments (Ali et al., 2023), proposed Faster-PestNet, a lightweight, MobileNet-based Faster R-CNN model. Designed to handle noise and lighting variations, it achieved 82.43% mAP on the IP102 dataset and over 95% accuracy on a smaller local dataset, outperforming standard detectors and proving effective for mobile field use.

The study proposes ESA-ResNet34 (Yuan et al., 2024), an enhanced lightweight deep learning model designed to address low accuracy, high complexity, and limited deployability in crop pest and disease detection. Using the challenging AI Challenger 2018 dataset, the model integrates Effective Spatial Attention, depthwise separable convolutions, and regularization to improve feature extraction and efficiency. ESA-ResNet34 achieves superior accuracy and significantly reduces parameters and FLOPs compared with existing architectures, making it suitable for mobile deployment. However, real-world variations such as blur and environmental noise remain challenging and highlight the need for further robustness improvements.

Also, in response to the broader challenge of limited generalizability in pest detection systems (Vilar-Andreu et al., 2024), proposes a unified insect presence detection approach using YOLOv8, wherein all insect types are grouped under a single generic class. This approach aims to overcome the over-specialization of existing models, making it more adaptable across diverse crops and environmental conditions. Leveraging the IP102 dataset, the YOLOv8-small model recorded a mAP@50 of 96.7% and a mAP@50–95 of 63.2%. The model’s strong accuracy and generalization capabilities highlight its potential as a scalable and practical solution for real-time agricultural pest monitoring.

In response to the urgent need for efficient monkeypox diagnosis, the study (Sun et al., 2024) introduces MpoxNet, a lightweight deep learning model based on the ConvNeXt architecture, designed for real-time clinical use. The model incorporates a dual-branch residual Squeeze-and-Excitation (D2RSE) module and a convolutional block attention module (CBAM) to enhance feature extraction and spatial focus while reducing complexity. Trained on the MSID dataset with data augmentation, MpoxNet achieved 95.28% accuracy and 95.80% F1-score, while maintaining only 30% of ConvNeXt-Tiny’s computational cost.

The study (Zhang et al., 2023) proposes an anchor-free detection model based on ConvNeXt-Tiny with FPN, RFEM, and BPA to address low contrast and complex backgrounds in infrared images. A dynamic soft label assignment improves localization. Tested on IIOPE and PASCAL VOC 2007, the model shows improved accuracy and generalization over anchor-based methods.

Vision Transformer (ViT) was introduced (Dosovitskiy et al., 2021), a novel architecture that replaces convolutional operations with pure Transformer encoders for image recognition. By dividing images into fixed-size patches (e.g., 16×16), linearly embedding them, and processing the resulting token sequence with positional encodings and a class token, ViT eliminates convolutional inductive biases. The model was pre-trained on large-scale datasets like ImageNet-21k and JFT-300M, then fine-tuned on benchmarks such as CIFAR-100, VTAB, and ImageNet-ReaL, achieving 88.55% top-1 accuracy on ImageNet and 94.55% on CIFAR-100. Results show that large-scale pretraining enables ViT to outperform or match state-of-the-art CNNs, especially with larger models and datasets.

The (Nobel et al., 2024) presents a hybrid deep learning approach for early and accurate detection of date palm leaf diseases, particularly those caused by Dubas insects and honeydew. It combines ECA-Net, ResNet50, and DenseNet201 with transfer learning to enhance feature extraction and classification. Trained on a curated, high-resolution dataset from the Aoun district in Iraq, the model classifies palm leaves into four categories: healthy, insect-infected, honeydew-infected, and both. Key steps include preprocessing, hybrid architecture design, ECA-based channel attention, and K-fold cross-validation. The proposed model achieved 99.54% training and 98.67% validation accuracy, outperforming individual models and showing strong generalization capabilities.

The study (Khan et al., 2024) addresses the limitations of existing plant disease detection systems, which often suffer from high computational cost, limited datasets, and suboptimal accuracy. Using the large and diverse PlantVillage dataset, the authors develop Bayesian-optimized hybrid deep learning models that integrate CNN-based feature extraction with classical machine-learning classifiers. The optimized CNN-Stacking model demonstrates superior performance and generalization, achieving over 98% accuracy on unseen data. While highly effective, future work is needed to extend the approach to more crop types and incorporate advanced localization techniques for improved practical deployment.

The study (Lin et al., 2024) addresses the limitations of existing plant disease recognition models, which often fail to capture both local and global symptom patterns and struggle with generalization across diverse field conditions. Using two large-scale datasets, the authors propose LGNet, a dual-branch architecture combining ConvNeXt-Tiny for local feature extraction and Swin Transformer-Tiny for global contextual learning, enhanced by adaptive feature fusion modules. The model achieves state-of-the-art performance with strong robustness across datasets, demonstrating superior discriminative capability. However, its dual-branch design increases computational complexity, highlighting the need for more lightweight solutions for real-world agricultural deployment.

(Liu et al., 2022) presents ConvNeXt, a modernized ConvNet architecture that integrates design elements from Vision Transformers into ResNet-like structures. This involves starting with a ResNet-50 baseline and applying systematic modifications, including hierarchical architecture, large kernel convolutions, inverted bottlenecks, and ResNeXt-style blocks. Training improvements include the use of AdamW optimizer and augmentations like Mixup, CutMix, and RandAugment. Each modification is validated through controlled experiments maintaining FLOPs and parameter budgets. The final ConvNeXt models are evaluated on ImageNet-1K, COCO, and ADE20K, achieving good results in classification, detection, and segmentation. The summary of the Literature Review is given in the **Table 1**.

**Table 1:** Comparative analysis of literature review on insect and pest detection.

Ref.	Journal, Year	Dataset	Technology	Results	Strengths	Limitations
(Hajjaji et al., 2025)	Ecological Informatics, 2025	349 aerial images (13,071 instances)	YOLOv5, YOLOv8	YOLOv8-HighAug: AP = 0.88, Precision = 0.87, Recall = 0.86	Evaluates multiple YOLO variants	Sensitive to background and lighting conditions
(Alnafisah and El-Shahed, 2024)	Alexandria Engineering Journal, 2024	Synthetic population model	Mathematical modeling (bifurcation)	Critical injection rate to eradicate RPW larvae	Supports long-term control planning	No cost or resource feasibility assessment
(Savaş, 2024)	Heliyon, 2024	Combined disease and pest datasets	Dirichlet Ensemble Neural Networks	DELM1 ROC AUC: 99%	Strong ensemble accuracy	High computational cost
(Hessane et al., 2023)	Big Data Mining and Analytics, 2023	2000 palm leaf images	SVM, KNN, RF, LightGBM	Acc: 98.3%	Addresses class imbalance	Requires high-quality annotations
(Chen et al., 2025)	Ecological Informatics, 2025	LP24, Pest24, VisDrone2019	DAMI-YOLOv8l	mAP@50: 78.2%, mAP@50:95: 57.3%, FPS: 121.12	Strong tiny-object detection	Requires manual hyperparameter tuning
(Sun et al., 2025)	IEEE Access, 2025	2090 UAV images (cotton crops)	YOLO-UP (YOLOv8n + modules)	+3.46% mAP, +5.16% Precision	SC3, AFPN, LSKA modules boost performance	Limited to RGB imagery
(Zhang et al., 2024)	IEEE Access, 2024	JutePest dataset (9 pest types)	JutePest-YOLO, YOLOv7 baseline	Precision: 98.7%, mAP@0.5: 95.68%	Improved model efficiency	No multi-frame tracking
(Ali et al., 2023)	IEEE Access, 2023	IP102 + Local Crop Dataset	Faster-RCNN + MobileNet	IP102: 82.43% mAP; Local: 95.24% Precision	Lightweight and benchmarked	Struggles with occlusion and tiny pests
(Vilar-Andreu et al., 2024)	IEEE Access, 2024	IP102 (102 classes)	YOLOv8	mAP@50: 0.967; mAP@50:95: 0.632	Scalable multi-crop framework	Lacks fine-grained species-level recognition
(Sun et al., 2024)	Frontiers in Cellular and Infection Microbiology, 2024	MSID dataset	MpoxNet (ConvNeXtbased)	Acc: 95.28%, F1: 95.8%	Efficient lightweight feature extraction	Limited real-world variance
(Zhang et al., 2023)	Energy Reports, 2023	IIOPE, VOC2007	ConvNeXt-Tiny + RFEM-BPA	Higher mAP across datasets	Strong multi-scale capability	Limited component ablation analysis
(Dosovitskiy et al., 2021)	ICLR, 2021	ImageNet, JFT-300M	Vision Transformer (ViT)	88.55% ImageNet Top-1	Excellent generalization	Requires large training datasets
(Nobel et al., 2024)	IEEE Access, 2024	Palm Leaves dataset	-Net, ResNet50, DenseNet201	Train: 99.54%, Val: 98.67%	Effective use of attention modules	Sensitive to dataset biases
(Khan et al., 2024)	Scientific Reports, 2024	PlantVillage (18,159 tomato leaf images, 10 classes)	Bayesian-optimized CNN + ML hybrid models	Accuracy: 98.52% (best model)	High accuracy, Robust generalization, efficient architecture	Limited to tomato leaves; needs extension and localization capabilities
(Lin et al., 2024)	Plant Phenomics, 2024	AI Challenger 2018 dataset	ConvNeXt-Tiny + Swin Transformer (dual-branch)	88.74%, 99.08%	High accuracy; strong local–global features	High complexity; limited real-world robustness

## 3 Materials and methods

**Figure 1:**
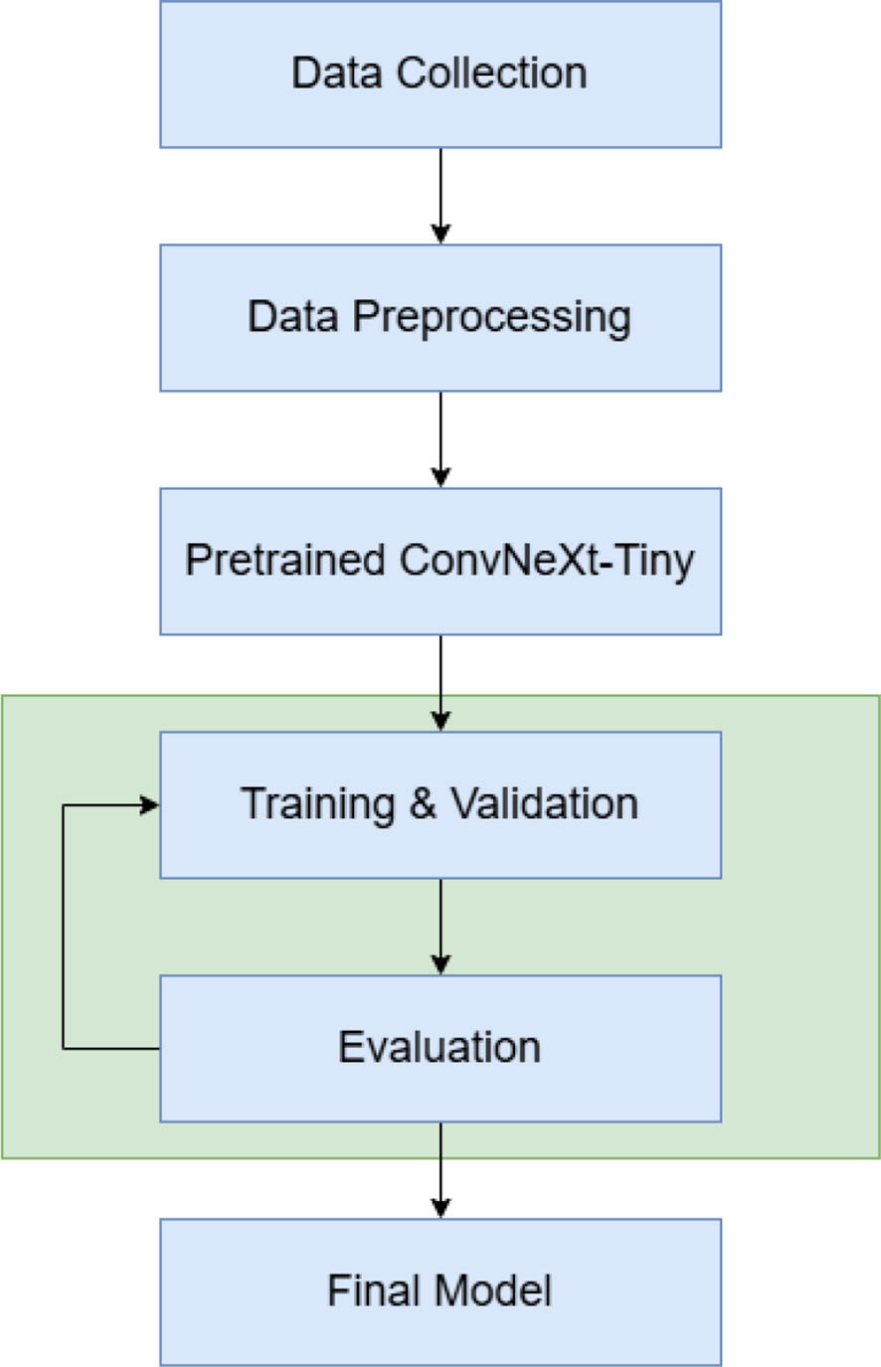
Workflow diagram of the proposed model.

The overall workflow of the proposed PalmNeXt is shown in **Figure 1**. The model follows a structured and efficient pipeline designed to ensure reliable and accurate pest classification from palm leaf images. The process begins with data collection, where RGB images of date palm leaves representing four categories, Bug, Dubas, Healthy, and Honey, are gathered from field conditions. This is followed by data preprocessing, which includes resizing, normalization, and preparing the dataset for model ingestion to enhance the quality and consistency of the input samples. The cleaned data is then fed into a pretrained ConvNeXt-Tiny backbone, leveraging transfer learning to extract robust and discriminative features relevant to pest identification. During the training and evaluation phase, the model learns class-specific patterns while performance is continuously monitored using validation metrics to prevent overfitting and ensure generalization. Afterward, the model undergoes a dedicated evaluation process on the unseen test set to assess its accuracy, precision, recall, F1-score, and behavior across classes through a confusion matrix. Finally, the best-performing model is saved as the final model, enabling deployment and further real-world application in precision agriculture. Each step of this workflow is explained in detail in the following sections.

### 3.1 Palm leaves dataset

In this study, we utilized the publicly available *Palm Leaves Dataset* provided by Warcoder on the Kaggle platform^1^. The dataset comprises a total of 4,000 high-resolution RGB images of palm leaves, systematically categorized into four distinct classes: *Bug*, *Dubas*, *Healthy*, and *Honey*. Each class represents a specific physiological or pathological condition of the palm leaves, enabling targeted classification and analysis. The images are organized into subdirectories based on their class labels and exhibit significant visual variability due to differences in lighting, leaf orientation, and background, thereby simulating real-world field conditions. This dataset provides a valuable resource for the development and evaluation of machine learning and deep learning models focused on plant disease detection, pest classification, and intelligent agricultural decision-making systems.

### 3.2 Data preprocessing

All images in the dataset underwent a standardized preprocessing pipeline implemented using the PyTorch transforms module. For the training set, a series of augmentation techniques were applied to enhance model generalization and reduce overfitting. Specifically, each image was resized to 224 × 224 pixels, followed by random horizontal flipping and random rotation up to 15^°^, enabling the model to learn invariance to orientation variations commonly observed in leaf images. The augmented images were then converted to tensors and normalized using a mean and standard deviation of [0.5, 0.5, 0.5]. In contrast, the validation and test sets were processed using only deterministic transformations, including resizing to 224 × 224, tensor conversion, and normalization, thereby ensuring consistent and unbiased evaluation of the model’s performance.

### 3.3 Proposed model

**Figure 2:**
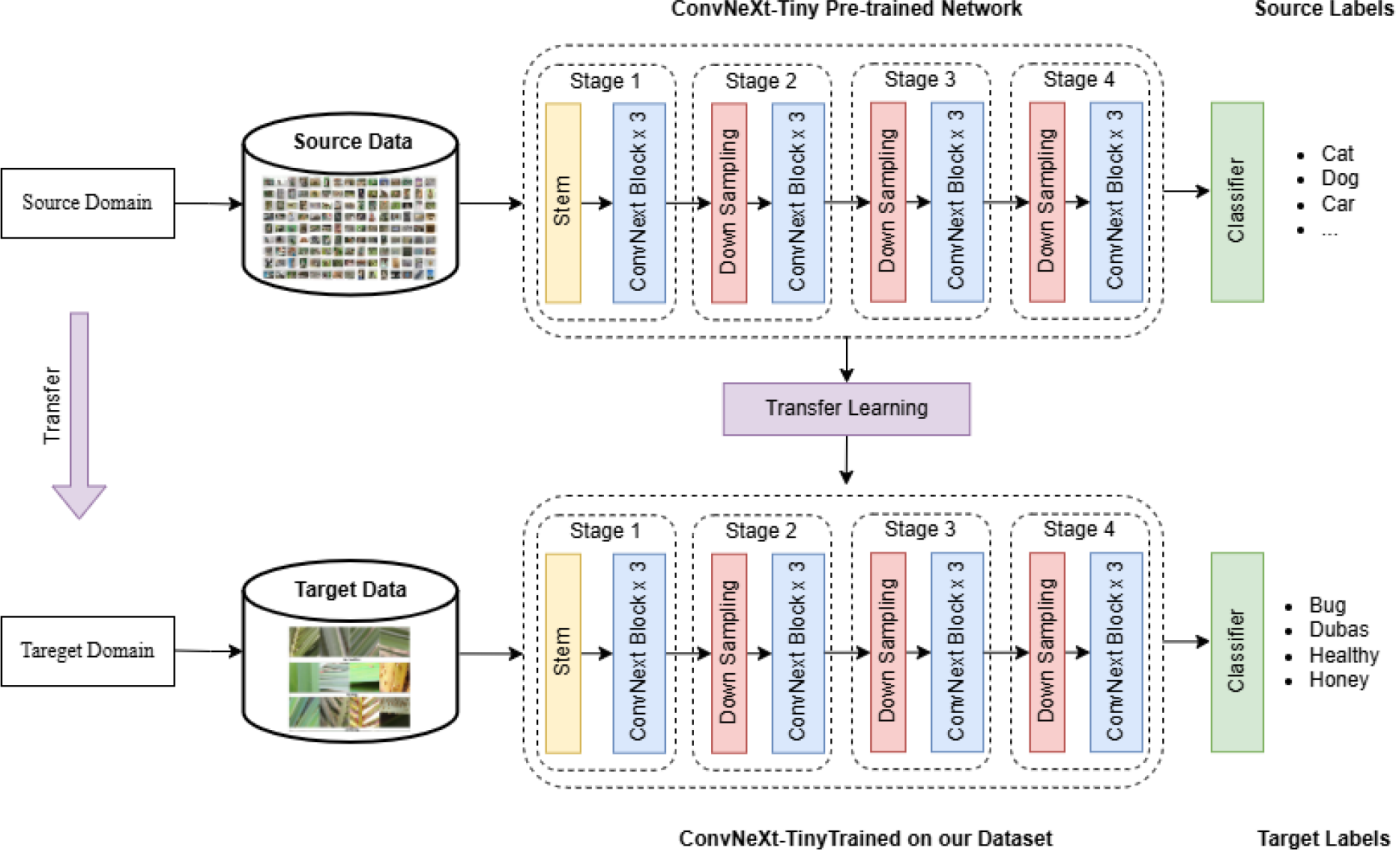
System architecture for palm leaf classification.

The architecture of the proposed model is illustrated in **Figure 2**, where transfer learning is employed by fine-tuning the ConvNeXt-Tiny network on our custom palm leaves disease dataset. Initially, the ConvNeXt-Tiny model is pre-trained on a large-scale source dataset (e.g., ImageNet) containing generic object categories such as cats, dogs, and vehicles (Liu et al., 2022). Leveraging this pre-trained knowledge, the model is adapted to the target domain comprising palm leaf images categorized into four classes: Bug, Dubas, Healthy, and Honey. The fine-tuning process allows the model to retain its learned low-level features while adjusting higher-level representations specific to the target task.

A detailed explanation of the ConvNeXt-Tiny architecture is provided in the subsequent section, highlighting its hierarchical structure, convolutional operations, and efficient design principles. The model comprises a stem layer followed by four stages, each containing multiple ConvNeXt blocks with depthwise separable convolutions and layer normalization. This enables the network to effectively capture both local and global contextual information from the input images, making it well-suited for fine-grained classification tasks such as disease detection in palm leaves.

#### 3.3.1 Input and patch embedding (stem layer)

The input image is denoted as $X\in {\mathbb{R}}^{H\times W\times C}$, where H and W represent the height and width of the image, respectively, and C denotes the number of color channels. In this work, we use input images of size *H* = *W* = 224 and *C* = 3. The stem layer is responsible for reducing the spatial resolution and increasing the depth of the feature map, acting as a patch embedding similar to the Vision Transformer (ViT).

This is achieved through a convolutional layer with a kernel size of 4 × 4 and stride 4, as shown in Equation 1:

(1) $$X_{0}={\text{Conv}}_{4\times 4,\text{ stride}=4}(X)$$

After this operation, the output feature map becomes:


$$X_{0}\in {\mathbb{R}}^{\frac{H}{4}\times \frac{W}{4}\times C^{\prime }},\text{ where }C^{\prime }=96$$

#### 3.3.2 ConvNeXt block

The ConvNeXt block extracts hierarchical features through an efficient convolutional structure composed of four key components: a depthwise convolution that captures local spatial information per channel, followed by channel-wise layer normalization to stabilize training. A pointwise feedforward network, implemented via two 1x1 convolutions with a GELU activation in between, then enriches the feature representations. Finally, a residual connection adds the original input to the output, preserving low-level information and ensuring robust gradient flow.

#### 3.3.3 Downsampling layer

To progressively reduce the spatial dimensions of feature maps between stages while increasing the channel depth, a convolutional layer with kernel size 2 × 2 and stride 2 is applied shown in Equation 2:

(2) $$z_{\text{down}}={\text{Conv}}_{2\times 2,\text{ stride}=2}(z)$$

This operation ensures that each subsequent stage captures higher-level semantic features at reduced spatial resolution.

#### 3.3.4 Stages overview

The ConvNeXt-Tiny model is structured into four hierarchical stages, with increasing numbers of channels and varying numbers of ConvNeXt blocks in each stage. This design allows for multi-scale feature learning. The ConvNeXt-Tiny architecture employs a four-stage hierarchical design, each contributing progressively to richer and more abstract feature representations. The initial stage captures low-level visual patterns such as edges and textures, while the subsequent stages focus on increasingly complex and semantic features. This gradual deepening of the network allows effective learning of both local and global context, which is particularly advantageous for fine-grained image classification tasks such as palm leaf disease detection. Moreover, the progressive downsampling across stages ensures a balanced trade-off between computational efficiency and model expressiveness, making ConvNeXt-Tiny both lightweight and accurate. The stage configuration is given in **Table 2**.

**Table 2:** ConvNeXt-Tiny stage configuration.

Stage	Blocks	Channels
1	3	96
2	3	192
3	9	384
4	3	768

#### 3.3.5 Global average pooling and classifier

The output from the final stage is processed using global average pooling (GAP) to reduce the spatial dimensions and create a fixed-length feature vector. This operation is given by Equation 3:

(3) $$z_{\text{avg}}=\frac{1}{HW}\sum_{i=1}^{H}\sum_{j=1}^{W}z_{i,j}$$

The resulting vector is passed through a fully connected layer followed by a Softmax activation to generate class probabilities as shown in Equation 4:

(4) $$\hat{y}=\text{Softmax}(Wz_{\text{avg}}+b)$$

Where $W\in {\mathbb{R}}^{K\times 768}$, $b\in {\mathbb{R}}^{K}$, and K is the number of classes.

#### 3.3.6 Loss function

To optimize the model during training, the categorical cross-entropy loss function is used as shown in Equation 5. This measures the divergence between predicted probabilities and true one-hot encoded labels:

(5) $$L_{\text{CE}}=-\sum_{i=1}^{K}y_{i}\text{log}({\hat{y}}_{i})$$

Where $y_{i}$ is the ground truth label and ${\hat{y}}_{i}$ is the predicted probability for class i. This loss function is minimized using stochastic gradient descent or Adam optimizer to improve classification performance.

## 4 Results

All experiments were conducted on the Kaggle platform utilizing dual NVIDIA T4 GPUs. The experiments were implemented using the Python programming environment with the PyTorch deep learning framework. The dataset was partitioned into three subsets: 75% for training, 15% for validation, and 10% for testing. The average inference time recorded for the proposed model was 7.182 ms per image, as measured on the specified hardware platform.

### 4.1 Implementation details

The ConvNeXt-Tiny architecture was employed as the backbone through transfer learning. The model was initialized with ImageNet-pretrained weights, and its classification head was modified to output four classes corresponding to the target categories. The entire network was fine-tuned end-to-end.

All images were preprocessed using the transforms module in PyTorch. The training dataset was augmented with standard techniques, including resizing, normalization, and random horizontal and vertical flips, to enhance model generalization. In contrast, the validation and test datasets underwent only deterministic preprocessing operations, such as resizing and normalization, to ensure a consistent evaluation of model performance.

Optimization was performed using the Adam optimizer with a learning rate of 1 × 10^−4^. A batch size of 32 was used for all data loaders. Cross-entropy loss served as the objective function. The model was trained for 50 epochs, and performance was evaluated at the end of each epoch on the validation dataset. For each epoch, the average training loss, validation loss, training accuracy, and validation accuracy were recorded to monitor convergence. The summary of the implementation setting is given in **Table 3**.

**Table 3:** Summary of implementation settings.

Component	Description/Value
Batch Size	32
Optimizer	Adam
Learning Rate	1 × 10^−4^
Loss Function	Cross-Entropy Loss
Training Epochs	50

### 4.2 Evaluation metrics

To evaluate the performance of the proposed classification model, standard evaluation metrics including Accuracy, Precision, Recall, and F1-Score were employed. These metrics are derived from the confusion matrix comprising true positives (TP), true negatives (TN), false positives (FP), and false negatives (FN).

• The Accuracy metric evaluates the overall correctness of the classification model. It is defined as the proportion of correctly classified samples relative to the total number of samples, as shown in Equation 6.

(6) $$\text{Accuracy}=\frac{TP+TN}{TP+TN+FP+FN}$$

• Precision evaluates the proportion of correctly predicted positive instances among all predicted positive instances, as shown in Equation 7.

(7) $$\text{Precision}=\frac{TP}{TP+FP}$$

• Recall (also known as Sensitivity) quantifies the proportion of actual positive cases that are correctly identified, as shown in Equation 8.

(8) $$\text{Recall}=\frac{TP}{TP+FN}$$

• F1-Score is the harmonic mean of Precision and Recall, and is used to balance the two in scenarios where class imbalance is present, as shown in Equation 9.

(9) $$\text{F}1-\text{Score}=2\times \frac{\text{Precision}\times \text{Recall}}{\text{Precision}+\text{Recall}}$$

These evaluation metrics provide a comprehensive understanding of the classification performance, especially under conditions of class imbalance.

### 4.3 Experimental results

To evaluate the performance of the models, experiments were conducted on the palm leaves dataset, which contains four categories: Bug, Dubas, Healthy, and Honey. The results for each class are presented below, comparing CNN + Attention, ResNet13 + Attention, ViT, and the proposed model.

#### 4.3.1 Results for bug class

The classification performance for the Bug class is summarized in **Table 4**. The proposed model achieved the highest recall and accuracy, significantly outperforming the other models in identifying bug-infected samples.

**Table 4:** Performance comparison for Bug class.

Model	Precision	Recall	F1-score	Accuracy
CNN + Attention	0.84	0.76	0.80	0.74
ResNet13 + Attention	0.79	0.73	0.76	0.78
ViT (Dosovitskiy et al., 2021)	0.86	0.79	0.82	0.70
ECA-Net (Nobel et al., 2024)	0.88	0.96	0.91	
ConvNeXt-Tiny (Liu et al., 2022)	0.40	0.69	0.50	0.56
Proposed Model	0.96	0.92	0.94	1.00

#### 4.3.2 Results for Dubas class

As shown in **Table 5**, the proposed model outperformed all other models, especially in precision and F1-score, indicating better handling of Dubas-infected samples despite class imbalance.

**Table 5:** Performance comparison for Dubas class.

Model	Precision	Recall	F1-score	Accuracy
CNN + Attention	0.61	0.67	0.63	0.74
ResNet13 + Attention	0.72	0.61	0.66	0.78
ViT (Dosovitskiy et al., 2021)	0.67	0.34	0.45	0.70
ECA-Net (Nobel et al., 2024)	0.83	0.80	0.81	
ConvNeXt-Tiny (Liu et al., 2022)	0.72	0.59	0.65	0.56
Proposed Model	0.86	0.94	0.90	0.99

#### 4.3.3 Results for healthy class

The Healthy class results, detailed in **Table 6**, demonstrate that the proposed model achieved perfect precision, recall, and accuracy, reflecting its robustness in identifying non-infected leaves.

**Table 6:** Performance comparison for healthy class.

Model	Precision	Recall	F1-score	Accuracy
CNN + Attention	0.90	0.95	0.92	0.74
ResNet13 + Attention	0.90	1.00	0.94	0.78
ViT (Dosovitskiy et al., 2021)	0.93	0.83	0.88	0.70
ECA-Net (Nobel et al., 2024)	0.99	0.99	0.99	
ConvNeXt-Tiny (Liu et al., 2022)	1.0	0.22	0.36	0.56
Proposed Model	1.00	0.99	0.99	0.1.00

#### 4.3.4 Results for honey class

**Table 7** presents the results for the Honey class, where the proposed model surpassed other methods across all metrics, especially F1-score and accuracy.

**Table 7:** Performance comparison for honey class.

Model	Precision	Recall	F1-score	Accuracy
CNN + Attention	0.66	0.61	0.63	0.74
ResNet13 + Attention	0.70	0.76	0.73	0.78
ViT (Dosovitskiy et al., 2021)	0.53	0.90	0.67	0.70
ECA-Net (Nobel et al., 2024)	0.85	0.82	0.83	
ConvNeXt-Tiny (Liu et al., 2022)	0.56	0.72	0.63	0.56
Proposed Model	0.95	0.90	0.92	0.99

#### 4.3.5 Cross validation performance

The 5-fold cross-validation results as shown in **Table 8**, demonstrate the consistent performance and generalization capability of the proposed model across multiple data splits. Training accuracy remained high for all folds, ranging from 95.54% to 97.42%, with an overall mean of 96.40% ± 2.35. This indicates that the model effectively learned discriminative features from the training data. Validation accuracy showed moderate variation across folds, ranging from 84.50% to 89.33%, with a mean of 86.63% ± 2.29, reflecting stable generalization despite the intrinsic variability within the dataset. The relatively small standard deviations in both training and validation accuracies further confirm the robustness of the model and its ability to maintain reliable performance across different subsets of the data. These results collectively validate the effectiveness of the proposed architecture in handling diverse input samples and reducing the risk of overfitting.

**Table 8:** 5-fold cross-validation performance metrics.

Fold	Train accuracy (%)	Validation accuracy (%)
Fold 1	96.67	88.83
Fold 2	97.42	85.83
Fold 3	96.54	84.50
Fold 4	95.54	89.33
Fold 5	95.87	84.67
Mean ± SD	96.40 ± 2.35	86.63 ± 2.29

#### 4.3.6 Overall comparison

The overall model performance across all classes is summarized in **Table 9**. The proposed model achieved the best results in every metric, validating its effectiveness for palm leaf classification tasks. The superior performance of the proposed model PalmNeXt over ConvNeXt-Tiny, Vision Transformer (ViT) and the hybrid ECA-Net (combining ResNet-50 and DenseNet-201) can be attributed to several key architectural advantages. First, unlike ViT which relies heavily on large-scale data and lacks strong inductive biases (e.g., locality and translation equivariance), ConvNeXt-Tiny retains the convolutional hierarchy that is inherently better suited for tasks with limited training data or requiring spatial locality, such as palm leaf disease classification.

**Table 9:** Overall performance comparison across all classes.

Model	Precision	Recall	F1-score	Accuracy
CNN + Attention	0.75	0.74	0.74	0.74
ResNet13 + Attention	0.77	0.78	0.77	0.78
ViT (Dosovitskiy et al., 2021)	0.73	0.72	0.71	0.70
ConvNeXt-Tiny (Liu et al., 2022)	0.68	0.56	0.54	0.56
Proposed Model	0.94	0.94	0.94	1.00

Second, ConvNeXt-Tiny benefits from modern design elements inspired by transformers, such as large kernel depthwise convolutions, GELU activations, and layer normalization, while preserving the efficiency and optimization stability of convolutional networks. Adding data preprocessing with these refinements result in stronger representation learning without requiring massive compute or data.

In contrast, ECA-Net, while leveraging the strengths of both ResNet and DenseNet backbones, introduces complexity and redundancy due to concatenation and feature fusion between architectures. This hybrid setup may lead to increased computational cost and a higher risk of overfitting, particularly on domain-specific datasets like palm leaf images. ConvNeXt-Tiny, on the other hand, offers a carefully balanced depth and parameter count, making it lightweight, efficient, and better generalized for fine-grained classification tasks.

PalmNeXt outperforms our custom lightweight CNN + Attention and ResNet13 + Attention models primarily due to its ConvNeXt-Tiny backbone, which incorporates a modernized convolutional design with improved feature extraction. Its hierarchical architecture, advanced normalization, and optimized convolutional blocks enable more robust learning of subtle visual patterns in palm leaf images. As a result, PalmNeXt achieves stronger representational capacity and better generalization, leading to superior classification performance.

**Figure 3:**
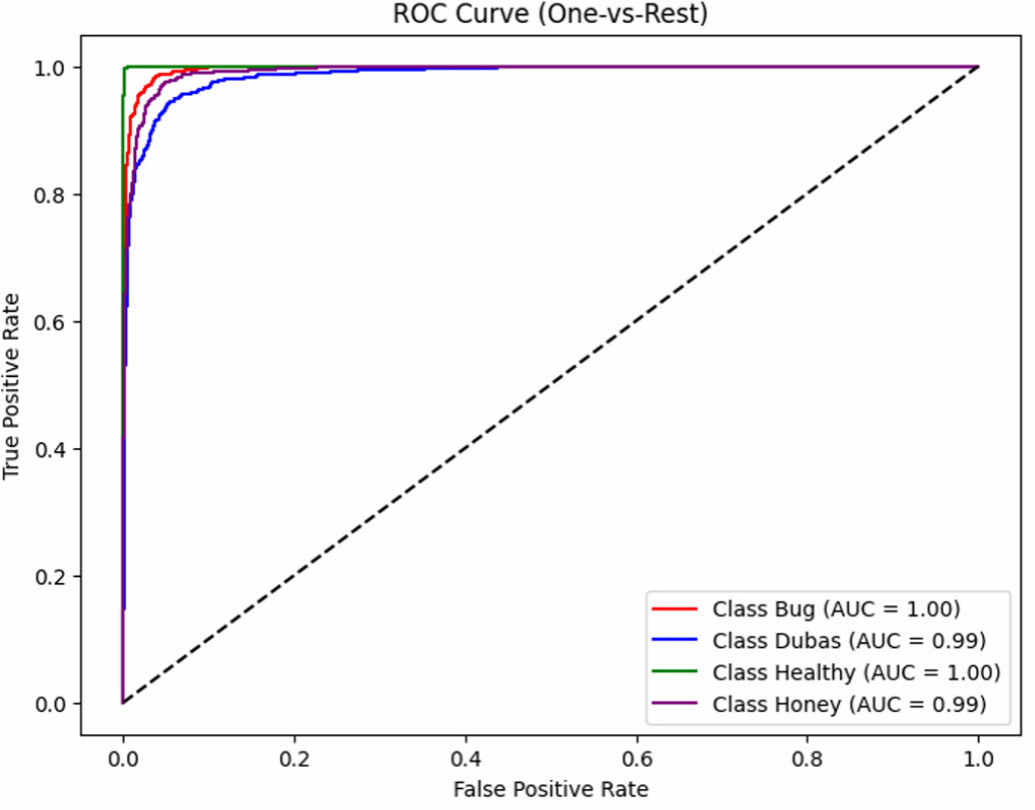
Receiver operating characteristic (ROC) curves for the four class classification of Bug, Dubas, Healthy, and Honey, using a one-versus-rest methodology.

The Receiver Operating Characteristic (ROC) curves for the four class classification system distinguishing between Bug infestation, Dubas infection, Healthy plants, and Honey secretion—demonstrate robust discriminatory performance across all categories. As illustrated in **Figure 3**, the multiclass ROC analysis, implemented through a one-vs-rest methodology, reveals area-under-curve (AUC) values of 1.00 for Bug, 0.99 for Dubas, 1.00 for Healthy, and 0.99 for Honey classification. The curves exhibit steep initial ascent and sustained high true positive rates across low false positive ranges, indicating strong model sensitivity with minimal type I errors. Notably, the Healthy and Bug class achieves near-perfect separability (AUC: 1.00), reflecting the model’s capacity to accurately distinguish unaffected specimens from pathological conditions. While all curves maintain AUC values above the random classifier baseline (0.5), the Honey and Dubas class presents a lower but still substantial discriminative capability (AUC: 0.99), suggesting slightly greater challenge in differentiating this physiological state from pathological manifestations. These collective metrics validate the model’s diagnostic precision across the complete phytopathological spectrum under investigation.

**Figure 4:**
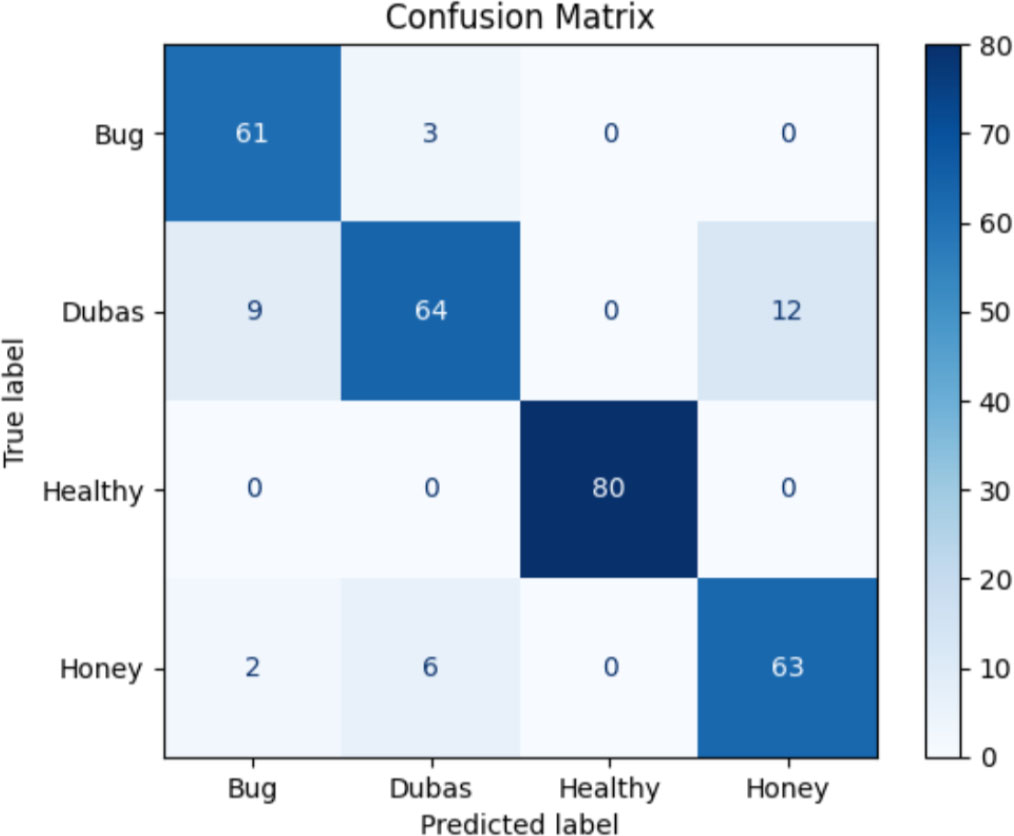
Confusion matrix of the proposed model results.

The confusion matrix reveals important insights into the classifier’s strengths and weaknesses across the four classes: Bug, Dubas, Healthy, and Honey, as shown in **Figure 4**. While the model performs well overall, several misclassification patterns highlight the inherent visual similarity of certain pest-related classes.

First, three Bug samples were misclassified as Dubas, suggesting that the morphological features of Bug-infected leaves share overlapping texture and color patterns with Dubas symptoms. This indicates that the model may rely heavily on global texture cues rather than fine-grained local patterns that differentiate these two pest categories.

Similarly, the Dubas class shows the highest confusion, with 9 samples classified as Bug and 12 as Honey. This dual-direction confusion implies class variability within Dubas images and potential intra-class inconsistency in the dataset. Dubas infection symptoms sometimes resemble Honeydew deposition due to similar yellowish discoloration, explaining misclassification toward the Honey class. The misclassification toward Bug may also stem from overlapping lesion shapes or lighting variations.

For the Honey class, 2 samples were misclassified as Bug and 6 as Dubas, reinforcing the earlier observation that Honeydew and Dubas share closely related visual characteristics. The confusion between Honey and Bug may also arise from shared background noise, leaf surface highlights, or subtle pest artifacts not easily distinguishable by the model.

Overall, the confusion matrix suggests that while PalmNeXt effectively learns general class boundaries, fine-grained discriminative feature learning should be improved, especially between pest-related classes with subtle visual differences. Future improvements may include incorporating attention-driven localization, class-balanced augmentation, or multi-scale feature enhancement to better capture minor symptom variations.

## 5 Discussion

The findings of this study highlight the effectiveness of the proposed PalmNeXt model in addressing the challenges of automated pest detection in date palm cultivation. By employing a lightweight ConvNeXt-Tiny backbone with transfer learning, the model reduces dependency on handcrafted features and traditional preprocessing pipelines, thereby improving computational efficiency and adaptability in real agricultural environments.

Overall, PalmNeXt delivered strong classification performance with high accuracy, precision, recall, and F1-score across all four pest classes. These results demonstrate the model’s capability to extract discriminative features despite variations in leaf texture, pest morphology, and image acquisition conditions. Furthermore, the model’s low computational cost reinforces its suitability for deployment in resource-limited agricultural settings.

A detailed examination of the confusion matrix provides additional insights into the model’s behavior on challenging samples. Specifically, 3 Bug samples were misclassified as Dubas, indicating visual overlap between these two pest categories. For the Dubas class, 9 samples were predicted as Bug and 12 as Honey, revealing that Dubas exhibits the highest confusion rate, likely due to fine-grained visual similarities with both classes and subtle color texture patterns. Similarly, Honey samples were occasionally misclassified: 2 as Bug and 6 as Dubas, which suggests that certain Honey features particularly color regions and leaf spots resemble those found in Bug and Dubas-affected leaves. These observations emphasize that some pest categories share morphological or visual characteristics that challenge deep learning models.

Future work will focus on enhancing robustness and scalability. This includes integrating class-balanced training strategies to reduce errors in highly confused classes like Dubas. Furthermore, we plan to explore advanced learning paradigms such as self-supervised learning on large volumes of unlabeled field imagery to learn more generalizable features and reduce annotation dependency. To ensure model reliability across diverse farms and conditions, investigating domain adaptation techniques will be a key direction. Finally, moving beyond RGB data, a promising avenue is multimodal sensing such as fusing RGB with thermal or hyperspectral imaging to capture discriminative features beyond the visible spectrum and improve separability among visually similar pests. Ultimately, deploying optimized versions of PalmNeXt on edge devices will be pursued for real-time, in-field monitoring.

This study contributes to precision agriculture by providing an accessible and scalable solution for early pest detection, enabling timely and informed interventions that minimize pesticide misuse and prevent large-scale crop loss. Future directions include integrating class-balanced training strategies, leveraging multispectral or hyperspectral imaging for improved separability among visually similar pests, and deploying PalmNeXt on edge devices for real-time, in-field monitoring.

## Data Availability

The original contributions presented in the study are included in the article/supplementary material. Further inquiries can be directed to the corresponding author.

## Footnotes

1
https://www.kaggle.com/datasets/warcoder/palm-leaves-dataset.
